# Supplementary material for: Surface Microbiome Profiling of Dental Elevators Using Third‐Generation Sequencing: Implications for Infection Control in Dental Practice
Source: Microbiologyopen. 2025 Dec 8;14(6):e70178. doi: 10.1002/mbo3.70178 (PMC12685768; doi:10.1002/mbo3.70178)
Supplement: Supplementary file 1 — customer_backup. [file MBO3-14-e70178-s001.zip › customer_backup/customer_backup/taxa_summary/krona/samples/M13.Krona.html]

Javascript must be enabled to view this page.

members
magnitude
magnitudeUnassigned

M13.krona

67516

67516

21

3

3

3

3

3

18

18

6

6

6

6

6

6

6

6

6

10998

370

370

28

17

17

2

2

5

5

4

3

1

342

300

66

234

42

42

46

23

23

1

1

6

6

3

3

11

11

2

2

1

1

1

1

22

16

15

15

1

1

1

1

1

2

2

2

3

1

1

2

2

13

10

1

1

1

9

1

1

8

8

3

2

2

1

1

1

1

1

10569

8963

8963

1377

1370

7

7586

229

98

44

198

6870

16

101

30

20

20

20

20

158

6

6

6

152

1

1

151

151

24

24

17

17

7

7

2

1

1

1

1

1

1

24

24

24

5

18

1

5

5

5

5

384

384

384

368

16

989

957

955

955

1

1

1

1

2

2

1

1

29

1

1

1

1

2

2

1

1

24

24

1

1

1

2

2

2

2

2

2

1

1

1

1

1

1

27

27

27

27

27

27

2560

2560

2560

156

2

2

154

154

2404

2404

1432

972

700

700

700

1

1

1

697

697

697

2

2

2

11

7

7

1

1

1

5

5

5

1

1

1

4

4

1

1

1

1

1

1

2

2

2

212

7

7

7

3

3

3

3

1

1

37

1

1

1

1

33

1

1

1

1

1

1

31

3

2

1

3

3

1

1

16

2

2

3

9

2

2

6

1

5

3

3

3

3

11

11

11

9

9

2

2

145

145

113

1

1

1

1

1

1

4

4

1

1

105

105

1

1

1

31

31

1

24

6

12

12

12

12

12

2

2

2

2

2

2

1

1

1

1

1

1

147

146

146

146

146

146

1

1

1

1

1

106

7

3

3

3

3

4

1

1

1

3

3

3

96

96

96

9

9

87

8

79

2

2

2

2

2

1

1

1

1

1

2

2

2

2

2

2

8

1

1

1

1

1

7

7

7

7

2

5

31

1

1

1

1

1

4

3

2

2

2

1

1

1

1

1

1

1

25

3

3

1

1

2

2

22

2

2

2

16

16

16

4

4

1

3

1

1

1

1

1

17

17

17

17

17

17

13767

659

1

1

1

1

259

259

1

1

1

1

1

1

1

1

255

99

155

1

1

1

1

1

221

221

1

1

220

220

106

1

1

1

4

4

3

1

4

3

3

1

1

28

1

1

27

27

17

5

5

1

1

3

2

1

3

3

1

1

4

1

2

1

1

1

1

51

1

1

48

48

2

2

1

1

1

1

7

7

7

2

2

3

63

63

1

1

1

1

59

1

58

1

1

1

1

13108

2

2

2

2

1

1

1

1

12072

1

1

1

1

1

1

92

2

2

90

90

7109

1273

1273

1

1

1

1

5365

5365

468

468

1

1

4

4

4

4865

4863

4863

1

1

1

1

344

104

53

53

46

46

5

5

1

1

1

1

1

1

235

36

36

199

199

2

2

2

1

1

1

259

259

257

219

38

2

2

20

20

20

6

14

1

1

1

1

1

1

1

1

146

135

135

135

11

11

11

7

1

1

1

2

2

2

4

4

4

1

1

1

1

10

5

5

5

5

1

1

2

2

2

1

1

3

3

3

3

4

4

4

1

3

1

1

1

1

236

1

1

1

10

10

7

3

5

1

1

4

4

1

1

1

219

1

1

218

213

5

1

1

1

1

1

1

23

14

14

14

14

1

13

9

7

6

6

6

1

1

1

2

2

2

2

2

2

2

2

2

2

37466

2603

2

2

2

1

1

3

3

3

3

403

7

6

6

1

1

396

1

1

395

395

10

10

10

10

1736

1735

7

7

5

5

8

5

1

1

1

3

3

16

4

12

4

4

1

1

57

28

29

1

1

149

149

71

70

1

85

3

1

80

1

30

30

814

814

1

1

2

2

1

1

1

1

1

1

47

1

39

7

213

213

1

1

18

18

7

7

192

1

93

18

1

22

9

41

4

1

2

1

1

1

172

172

2

2

170

170

143

140

140

140

3

3

3

100

71

2

2

2

2

2

2

3

1

2

3

3

2

1

1

1

1

5

5

3

3

48

45

3

6

6

6

19

1

1

3

2

1

9

9

1

1

5

1

3

1

4

3

3

1

1

34

34

34

34

19245

93

93

92

91

1

1

1

1

1

1

1

322

99

1

1

3

3

95

91

4

223

223

219

4

1

1

1

1

16791

3

3

3

244

244

244

13950

13950

8

1633

81

1051

2938

341

2

7709

185

2

2

2

2

2583

1240

328

90

822

31

31

472

472

2

2

780

779

1

4

3

1

37

37

9

9

8

6

1

1

9

9

9

773

3

2

2

1

1

770

1

1

769

2

1

323

207

236

1264

30

1

1

9

9

12

7

5

3

3

3

1

2

2

2

1234

638

638

151

151

362

362

5

5

7

7

59

59

5

1

4

3

3

2

2

2

2

15618

15618

1052

1

1

1051

807

150

94

14565

14540

14540

25

25

1

1

1

185

185

185

185

185

185

1216

1

1

1

1

1

1207

126

1

1

1

125

125

125

122

91

89

89

1

1

1

1

31

31

31

883

2

2

2

137

3

3

26

26

108

108

3

3

3

5

5

2

2

1

2

1

1

1

1

6

6

2

4

1

1

1

13

13

12

1

66

66

2

22

25

3

12

2

143

5

5

125

125

13

3

2

3

1

4

505

8

8

161

137

24

336

69

267

76

1

1

1

1

1

1

63

63

63

11

11

11

3

3

3

3

3

5

5

2

2

2

3

1

1

2

2

4

4

1

1

1

1

2

2

2

2

1

1

1

1

1

1

1

1

1

1

5

5

5

5

4

4

1

1
